# Antimicrobial Efficacy of Nd:YAG Laser in Polymicrobial Root Canal Infections: A Systematic Review of In Vitro Studies

**DOI:** 10.3390/ijms26125631

**Published:** 2025-06-12

**Authors:** Jakub Fiegler-Rudol, Dariusz Skaba, Rafał Wiench

**Affiliations:** Department of Periodontal Diseases and Oral Mucosa Diseases, Faculty of Medical Sciences in Zabrze, Medical University of Silesia, 40-055 Katowice, Poland; rwiench@sum.edu.pl

**Keywords:** Nd:YAG laser, root canal disinfection, polymicrobial infection, antimicrobial efficacy, molecular mechanisms, photothermal therapy, biofilm eradication, endodontic pathogens, dentinal tubules, laser-assisted endodontics

## Abstract

Endodontic infections are characterized by complex polymicrobial communities residing within the intricate root canal system. Traditional chemomechanical methods frequently fail to achieve complete microbial eradication, especially in cases involving biofilm-forming and resistant species. This systematic review synthesizes current evidence on the molecular basis and antimicrobial efficacy of the neodymium-doped yttrium aluminum garnet (Nd:YAG) laser in root canal disinfection, particularly against polymicrobial infections. A comprehensive literature search was conducted in the PubMed, Embase, Scopus, and Cochrane databases in accordance with PRISMA 2020 guidelines. Experimental and preclinical studies evaluating the bactericidal properties of Nd:YAG laser therapy were included. The Nd:YAG laser demonstrated significant reductions in total microbial load through photothermal effects, including denaturation of proteins, disruption of cell membranes, and degradation of mixed-species biofilms. Although complete sterilization was not consistently achieved, its ability to penetrate dentinal tubules and target microbial consortia offers substantial adjunctive value. Standardization of laser parameters and further clinical studies are needed to validate these findings and establish Nd:YAG laser use in routine endodontic disinfection protocols.

## 1. Introduction

Successful endodontic therapy fundamentally depends on the thorough and effective elimination of microbial pathogens from the intricate and often inaccessible areas of the root canal system [1,2,3,4]. Despite significant progress in mechanical instrumentation and the development of sophisticated chemical irrigation techniques, achieving complete and sustained disinfection of the complex root canal anatomy continues to pose a formidable clinical challenge for practitioners [5,6]. This difficulty is primarily due to the presence of anatomical complexities such as lateral canals, isthmuses, and apical deltas that harbor resilient microbial populations beyond the reach of traditional treatment modalities [7]. Among these microbial species, *Enterococcus faecalis* is particularly notorious for its resistance to eradication [8]. This facultative anaerobe exhibits an extraordinary ability to survive under nutrient-deprived conditions and to deeply infiltrate the dentinal tubules, forming persistent biofilms that are impervious to conventional chemomechanical protocols [9,10]. Its survival in treated canals has been closely associated with endodontic treatment failures, thus highlighting the need for innovative disinfection strategies [11,12]. In response to these challenges, there has been a growing interest in the development and clinical integration of adjunctive technologies that can augment the antimicrobial efficacy of existing methods [13]. One such promising approach involves the application of laser-based systems, with particular attention being given to the neodymium-doped yttrium aluminum garnet (Nd:YAG) laser [14]. This laser, which operates at a wavelength of 1064 nanometers, exerts its primary antimicrobial effects through photothermal interactions, leading to localized heat generation capable of penetrating deeply into the dentinal tissues [15,16]. Unlike conventional irrigants, which often struggle to reach the depths of the dentinal tubules, the Nd:YAG laser offers superior tissue penetration, potentially enhancing its ability to eliminate deeply embedded microorganisms [17]. Furthermore, the Nd:YAG laser has demonstrated the capacity to disrupt bacterial cell walls and degrade biofilm matrices, making it an attractive candidate for endodontic disinfection, particularly in recalcitrant cases where standard treatment approaches prove insufficient [18,19,20]. A growing body of in vitro and ex vivo research has investigated the bactericidal potential of the Nd:YAG laser, frequently comparing its performance with that of other laser systems such as the erbium-doped yttrium aluminum garnet (Er:YAG) and erbium, chromium-doped yttrium, scandium, gallium, and garnet (Er,Cr:YSGG) lasers, as well as with commonly used chemical irrigants like sodium hypochlorite and chlorhexidine [21,22,23,24,25]. In addition, some studies have explored its utility alongside emerging methods such as antimicrobial photodynamic therapy [26]. However, despite these encouraging findings, results across individual studies have been inconsistent and sometimes contradictory [27,28]. Variability in experimental methodologies, including differences in laser energy settings, pulse durations, microbial strains targeted, and methods of microbial quantification, have contributed to a lack of consensus in the literature [29,30,31,32]. This inconsistency is further compounded by the predominance of laboratory-based investigations and the relative scarcity of well-designed clinical trials, which limits the generalizability of findings to real-world clinical settings [33]. Moreover, the absence of standardized treatment protocols impedes the reproducibility and comparability of outcomes across studies. Given these limitations and the need for evidence-based clinical guidance, there is a critical imperative to systematically evaluate and synthesize the available evidence regarding the antimicrobial efficacy of the Nd:YAG laser as an adjunctive root canal disinfectant.

The primary objective of this systematic review is to collate and critically appraise microbiological outcomes reported in the existing body of literature, with a particular focus on measures such as bacterial eradication rates and reductions in microbial load. By undertaking a rigorous analysis of recent experimental and preclinical studies, this review seeks to clarify the potential role of Nd:YAG laser therapy in enhancing endodontic disinfection. Ultimately, the findings aim to inform future research directions and support the development of standardized clinical protocols for the effective integration of laser technology into routine endodontic practice.

## 2. Methods

### 2.1. Focused Question

A systematic review was conducted using the PICO framework [34], structured as follows: infected root canal system models (Population), does treatment with the Nd:YAG laser (Intervention), compared to conventional antimicrobial therapies, alternative laser modalities, or no laser treatment (Comparison), result in improved bacterial eradication or reduction of microbial load (Outcome)?

### 2.2. Search Strategy

This systematic review, registered with PROSPERO (ID: CRD420251038941), was conducted in accordance with the PRISMA 2020 guidelines to ensure transparent and structured reporting [35]. This review followed PRISMA 2020 guidelines and included a clearly defined focused question (PICO), search strategy, selection criteria, risk of bias assessment, and data extraction protocols to ensure methodological transparency and reproducibility. A comprehensive literature search was performed across major electronic databases, including PubMed/Medline, Embase, Scopus, and the Cochrane Library, to identify studies evaluating the antibacterial and bactericidal efficacy of the Nd:YAG laser in root canal disinfection. The complete search strategy is illustrated in Section 3.1. Three independent reviewers executed the database queries using a predefined set of search terms focused on microbiological outcomes associated with Nd:YAG laser use. Language restrictions were applied to include only studies published in English without date restrictions. The study selection process began with title and abstract screening based on established inclusion criteria (outlined in Table 1), followed by independent full-text assessments conducted by two reviewers. A snowballing technique was also employed, reviewing the reference lists of included articles to identify additional eligible studies. The primary aim of this review was to synthesize available microbiological evidence on the effectiveness of Nd:YAG laser therapy in reducing or eliminating bacterial pathogens within the root canal system, whether used alone or as an adjunct to conventional disinfection protocols. Final study inclusion was based on strict adherence to predefined eligibility criteria. The literature search was conducted from database inception to February 2025.

### 2.3. Study Selection Process

To ensure methodological rigor and minimize potential bias, all identified records underwent a structured and independent screening process carried out by multiple reviewers. Titles and abstracts were systematically evaluated for consistency with predefined eligibility criteria. In cases of disagreement, consensus was reached through discussion among reviewers to maintain uniform decision-making standards. The selection criteria were specifically designed to identify high-quality experimental studies assessing the antimicrobial efficacy of the Nd:YAG laser in root canal disinfection (Table 2).

This evaluation, conducted in alignment with PRISMA 2020 guidelines, was designed to ensure transparency, reproducibility, and methodological integrity. Only studies demonstrating clear relevance and scientific rigor were selected for inclusion. This stringent selection process was implemented to generate a credible and evidence-based synthesis of current microbiological data on the antibacterial and bactericidal efficacy of the Nd:YAG laser in the context of root canal disinfection.

### 2.4. Risk of Bias in Individual Studies

To promote impartiality and reduce the likelihood of selection bias, the initial screening of titles and abstracts retrieved from the search strategy was conducted independently by three reviewers. To quantify the consistency of their evaluations, the Cohen’s kappa coefficient was calculated, offering an objective measure of interviewer agreement [36]. When disagreements arose concerning the eligibility of specific studies, they were addressed through deliberate, structured dialogue until unanimous agreement was achieved. This coordinated and transparent process was designed to preserve the methodological integrity of the review and ensure a reliable selection of relevant studies.

### 2.5. Quality Assessment

The methodological quality of the included studies was independently assessed by three reviewers, with a focus on key elements related to the design, execution, and reporting of Nd:YAG laser interventions. A structured scoring system was used to evaluate risk of bias, assigning 1 point for each criterion met (“yes”) and 0 points for unmet items (“no”), across nine predefined domains: (1) clear reporting of Nd:YAG laser operating parameters (e.g., power settings, frequency, pulse duration); (2) identification of the laser device or manufacturer; (3) comprehensive description of the irradiation protocol, including exposure duration and treatment area; (4) provision of full technical specifications such as wavelength, spot size, energy fluence, and repetition rate; (5) use of dosimetric validation tools (e.g., power meter); (6) inclusion of an appropriate control group (e.g., untreated, placebo, or comparative antimicrobial treatment); (7) application of valid statistical analysis for microbiological outcomes; (8) transparent outcome reporting without selective or missing data; and (9) disclosure of conflicts of interest and funding sources. Each study received a total score out of 9, and risk of bias was categorized as high (0–3 points), moderate (4–6 points), or low (7–9 points). Final quality ratings were made in accordance with the Cochrane Handbook for Systematic Reviews of Interventions [37] (Table 3).

### 2.6. Data Extraction

Once a consensus was reached on the final list of studies for inclusion, two reviewers independently extracted data using a predefined and standardized protocol to promote uniformity and minimize errors. The extracted information encompassed key study characteristics, including bibliographic details (first author, year of publication), study design, microbial species or strains evaluated, descriptions of experimental and control groups, follow-up duration where applicable, and the primary and secondary microbiological outcomes assessed. Particular attention was given to comprehensive technical details related to the Nd:YAG laser intervention, such as wavelength, power output, pulse frequency, energy fluence, and fiber type, as well as any adjunctive agents or methods used in conjunction with the laser. Procedural variables, including treatment duration, exposure technique, and application mode, were also systematically recorded to facilitate cross-study comparison and synthesis.

## 3. Results

### 3.1. Study Selection

**Figure 1:**
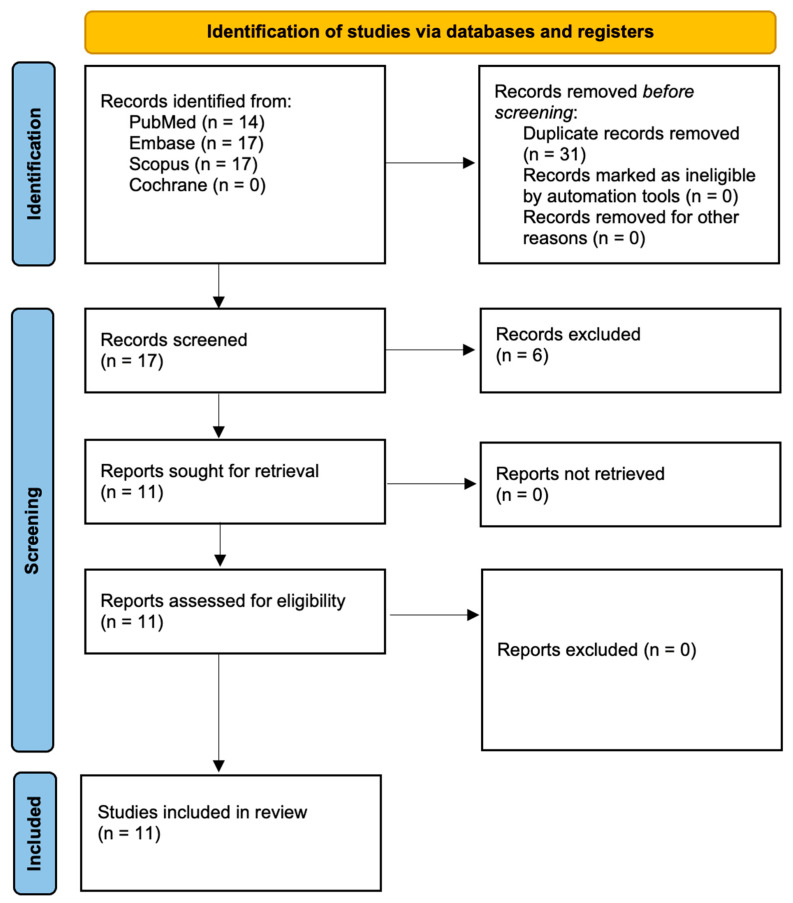
PRISMA 2020 flow diagram.

In alignment with the PRISMA 2020 guidelines, the study selection process is summarized in Figure 1. The initial database search retrieved 48 records, which were reduced to 17 distinct entries following the removal of duplicates. A preliminary screening of titles and abstracts confirmed the relevance of 11 studies, which were subsequently subjected to full-text review. No studies were excluded at this stage, resulting in the final inclusion of 11 publications for analysis. These studies were selected based on their direct investigation of the antimicrobial and bactericidal properties of the Nd:YAG laser in endodontic disinfection.

### 3.2. Data Presentation

Table 4, Table 5, Table 6 and Table 7 present a detailed and organized synthesis of the results from the ten included studies, highlighting essential outcomes, methodological characteristics, and microbiological data pertaining to the antibacterial and bactericidal properties of the Nd:YAG laser.

### 3.3. Overview of Study Characteristics

Table 4 outlines the geographical distribution of the studies included in this review.

**Table 4:** The geographical origins of the included research.

Study	Country
Bergmans et al., 2006 [38]	Belgium
Cheng et al., 2012 [39]	China
Jurić et al., 2016 [40]	Croatia
Kasić et al., 2017 [41]	Croatia and France
Katalinić et al., 2014 [42]	Croatia and Austria
Meire et al., 2012 [43]	Belgium
Moshonov et al., 1995 [44]	USA
Pirnat et al., 2011 [45]	Slovenia
Schoop et al., 2004 [46]	Austria
Vatkar et al., 2016 [47]	India
Wang et al., 2018 [48]	China

### 3.4. Main Study Outcomes

The outcomes of the included studies are summarised in Table 5.

**Table 5:** Summary of principal results and study details.

Reference Number	Author and Year	Microorganisms Tested	Study Groups	Outcomes
[38]	Bergmans et al., 2006	*Enterococcus faecalis* (LMG 7937), *Streptococcus anginosus*, and *Actinomyces naeslundii*	The experimental design included three main models. In the microbiological analysis, six root canals were inoculated with *E. faecalis* and divided into laser-treated and control groups, using a cross-over approach. For CSEM, dentine discs were inoculated with *S. anginosus* and *A. naeslundii*, then treated with one, two, or three cycles of laser irradiation through 1 mm thick dentine. ESEM was employed to assess the effect of direct laser irradiation on biofilms formed by *E. faecalis* or micro-colonies of *S. anginosus*, with specimens undergoing either two or three irradiation cycles and being analyzed in situ.	- Nd:YAG laser reduced *E. faecalis* load in root canals by 99.7%. - CSEM showed increasing bacterial damage with each laser cycle, with *A. naeslundii* more susceptible than *S. anginosus*. - No intact cells remained after three cycles. - ESEM revealed that while *S. anginosus* micro-colonies were mostly eradicated after 2–3 cycles, *E. faecalis* biofilms remained partially intact, with viable bacteria persisting in deeper layers, underscoring the difficulty of eliminating biofilm-associated infections.
[39]	Cheng et al., 2012	*Enterococcus faecalis* (ATCC 4083)	The study comprised seven groups: five experimental groups—(1) Nd:YAG laser, (2) Er:YAG/NaClO/NS/DW, (3) Er:YAG/NS/DW, (4) Er,Cr:YSGG laser, and (5) aPDT—and two control groups: 5.25% NaClO as the positive control and 0.9% normal saline as the negative control. Each group included 20 specimens for bacteriological analysis and 10 for SEM imaging. Laser settings and procedural specifics followed manufacturer guidelines.	- All laser protocols significantly reduced bacterial loads, with the Er:YAG/NaClO/NS/DW group achieving complete elimination on canal walls and up to 200 μm into dentinal tubules. - While 5.25% NaClO achieved 99.99% surface reduction, it was less effective in deeper layers. - Nd:YAG, Er,Cr:YSGG, and aPDT showed strong but lower efficacy. SEM confirmed no intact bacteria in Er:YAG/NaClO/NS/DW samples at all depths, unlike other groups. - The study concluded this combination was the most effective for endodontic disinfection.
[40]	Jurić et al., 2016	*Enterococcus faecalis* (ATCC 29212)	A total of 65 dentine samples were randomly assigned to five groups: aPDT using phenothiazinium chloride and 660 nm laser; Nd:YAG laser irradiation at 2 W, 15 Hz; QMiX solution (a mix of CHX, EDTA, and detergent); 5.25% NaOCl as the negative control (gold standard disinfectant); and a positive control group with no disinfection. Each group included 15 samples for quantitative and microscopic analysis.	- All methods significantly reduced *E. faecalis* CFUs versus controls. - The most effective was 5.25% NaOCl (99.99% reduction; complete eradication in some cases), followed by QMiX (99.3%) and aPDT (98.8%) with similar efficacy. Nd:YAG laser was least effective (96% reduction), with FISH confirming residual viable biofilm. - Overall, QMiX and aPDT were effective NaOCl alternatives, while laser alone showed limited efficacy.
[41]	Kasić et al., 2017	*Enterococcus faecalis* and *Candida albicans*	Thirty extracted single-rooted human teeth were divided into three groups (n = 10), each disinfected with a different laser system plus saline irrigation. Group 1 received Er:YAG laser using the PIPS technique, Group 2 underwent Nd:YAG laser treatment with spiral fiber motion, and Group 3 was treated with Er,Cr:YSGG laser using a radially firing tip. CFUs were measured before and after treatment to evaluate antimicrobial effectiveness.	- The Er,Cr:YSGG laser most effectively reduced *E. faecalis* and *C. albicans*, significantly outperforming Er:YAG and Nd:YAG (*p* < 0.05). Er:YAG also reduced CFUs significantly, but less so. Nd:YAG showed no statistically significant effect. - These results highlight erbium lasers, especially Er,Cr:YSGG, as better suited for root canal disinfection due to superior irrigant activation and deeper penetration in biofilm-rich environments.
[42]	Katalinić et al., 2014	*	Sixty extracted single-rooted anterior teeth were divided into four groups (n = 15) based on the final disinfection method: (1) 2.5% NaOCl, (2) 0.2% CHX, (3) gaseous ozone, and (4) Nd:YAG laser. After disinfection, canals were filled, post spaces prepared, and fiber-reinforced composite posts cemented with a self-etch adhesive. Push-out bond strength was measured using a universal testing machine.	- Nd:YAG laser treatment produced the highest bond strength values, significantly outperforming the NaOCl group (*p* = 0.004). Sodium hypochlorite irrigation resulted in the lowest bond strength, potentially due to its oxidizing effect on dentine. No significant differences were found between the CHX, ozone, and Nd:YAG groups, nor between CHX, ozone, and NaOCl. The findings suggest that laser treatment enhances bonding effectiveness, while NaOCl may compromise it.
[43]	Meire et al., 2012	*Enterococcus faecalis* (ATCC 10541), *Candida albicans* (ATCC 10231), and *Propionibacterium acnes* (LMG 16711)	Two laser systems were tested in vitro: Er:YAG (2940 nm) in single-pulse mode with energies from 40–400 mJ and pulse durations of 100–1000 μs, and Nd:YAG (1064 nm) in pulse train mode at 15 W, 100 Hz, applied for 5–120 s. Microbe-inoculated agar plates were irradiated through a 5 mm spot; for Nd:YAG, spot size was varied to assess irradiance effects. Antimicrobial efficacy was evaluated by measuring inhibition or clear zone diameters post-irradiation.	- Er:YAG laser effectively inhibited all tested microorganisms on agar at low energy thresholds (TITs ≤ 220 mJ), with efficacy varying by pulse length and species, *P. acnes* being most sensitive. Shorter pulses worked better for bacteria. - In contrast, Nd:YAG required much higher energy densities (e.g., 5300 J/cm^2^ for *C. albicans*, 7100 J/cm^2^ for *P. acnes*) and was ineffective against surface *E. faecalis*, though embedded cells showed moderate susceptibility. - Overall, Er:YAG offered superior surface antimicrobial action, while Nd:YAG showed deeper but less efficient photothermal effects.
[44]	Moshonov et al., 1995	*Enterococcus faecalis*	Seventy-five teeth were assigned to six groups: (1) non-infected control, (2) infected control, (3) infected + Nd:YAG laser with nigrosin dye, (4) infected + dye only, (5) infected + dye and air-water spray (no laser), and (6) infected + 1% NaOCl (positive control). In Group 3, laser irradiation was applied using a 400 µm fiber at 4.5 W, alternating between apical and coronal directions. Post-treatment, all teeth were sampled for bacterial growth, and selected specimens underwent SEM analysis.	- Nd:YAG laser treatment reduced cultivable *E. faecalis* by over 99%, indicating strong but incomplete disinfection. - NaOCl achieved complete elimination. SEM confirmed partial canal wall cleaning and smear layer reduction with some residual bacteria. - Dye and air-water spray controls showed minimal effect, highlighting the laser’s specific antibacterial action. - Nd:YAG demonstrated notable potential, though less effective than NaOCl in achieving full sterility.
[45]	Pirnat et al., 2011	*Enterococcus faecalis* (ATCC 29212)	The experiment tested two Nd:YAG laser heating protocols: (1) single 25 ms pulses (60–100 J/cm^2^) and (2) pulse trains of ten 1 ms pulses with 30 ms intervals (total fluence 100–260 J/cm^2^). Bacterial viability was assessed via culture plates and flow cytometry. Thermal behavior and disinfection efficacy were also compared between healthy and carious dentin using modeled heat pulses with fluences from 30 to 300 J/cm^2^.	- Viability of *E. faecalis* decreased significantly with increasing peak temperature and laser fluence. - A ≥90% reduction (1-log_10_) in viability was achieved at around 102 °C with an 8 ms effective exposure time. - Sub-millisecond pulse trains produced cumulative thermal effects comparable to longer pulses, and the effectiveness was modeled accurately with the Arrhenius-based TDSC. - Disinfection was more efficient in carious than in healthy dentin due to higher absorption. - The study concluded that sub-millisecond pulsed Nd:YAG lasers are more effective and energy-efficient for root canal disinfection than continuous-wave lasers, minimizing unwanted thermal damage.
[46]	Schoop et al., 2004	*Escherichia coli* (ATCC 25922), *Enterococcus faecalis* (ATCC 29212)	Four dental lasers were tested: Nd:YAG (1064 nm), diode (810 nm), Er:YAG (2940 nm), and Er,Cr:YSGG (2780 nm), each at 1 W and 1.5 W, forming eight test groups plus controls. Each group included 20 dentin slices. Laser irradiation was applied indirectly through the non-inoculated side to simulate clinical deep-layer disinfection. Control groups received no irradiation. Temperature measurements were also recorded to evaluate thermal effects.	- All four lasers showed bactericidal effects, strongest at 1.5 W, with *E. coli* more easily eradicated than *E. faecalis*. - Er:YAG was most effective, fully eliminating *E. coli* and reducing *E. faecalis* by up to two log steps. - Diode and Er,Cr:YSGG were less effective, especially against *E. faecalis*. Nd:YAG and Er:YAG outperformed others in both reduction and penetration. - Temperature rises were moderate (max ~8.7 °C) and unrelated to efficacy. - Overall, all lasers were effective for deep dentin disinfection, with Er:YAG showing the highest efficiency.
[47]	Vatkar et al., 2016	*Enterococcus faecalis* (ATCC 29212)	Group I (control) received no disinfection, allowing assessment of natural bacterial colonization. Group II was irrigated with 0.9% saline, Group III with 5.25% NaOCl, and Group IV with 2% CHX. Groups V and VI underwent laser disinfection using Nd:YAG and diode lasers, respectively, with specified power settings and fiber-optic delivery.	- The control group showed deep *E. faecalis* colonization (965–1176 µm). - Normal saline was ineffective, with no zone of dead bacteria (ZDB). NaOCl and CHX produced limited ZDBs (88–110 µm and 110–194 µm, respectively). - In contrast, Nd:YAG and diode lasers achieved complete bacterial eradication, with ZDBs spanning nearly the full dentin depth (761–1145 µm). - Laser methods were markedly more effective than traditional irrigants in disinfecting dentinal tubules.
[48]	Wang et al., 2018	*Enterococcus faecalis* (ATCC 29212)	Specimens were randomly divided into six groups, each tested at 1 and 3 min intervals: (A) 5.25% NaOCl, (B) Nd:YAG laser, (C) diode laser, (D) Nd:YAP laser, (E) Er,Cr:YSGG + NaOCl, and (F) Er:YAG + NaOCl. Bactericidal effects were evaluated using CLSM with LIVE/DEAD staining, comparing outcomes across laser types and exposure times.	- All treatments significantly reduced E. faecalis biofilms, with greater efficacy at 3 min. - Er:YAG + NaOCl and Er,Cr:YSGG + NaOCl showed the highest bactericidal activity (76–89% and 73–85% cell death), outperforming all other groups. - Nd:YAP was next most effective, while Nd:YAG and diode lasers were less effective but still better than NaOCl alone. - The study emphasized the synergistic effect of erbium lasers with NaOCl, enhancing disinfection via photoacoustic and cavitation mechanisms for deeper irrigant penetration.

CSEM—conventional scanning electron microscopy, ESEM—environmental scanning electron microscopy, Nd:YAG—neodymium-doped yttrium aluminum garnet, Er:YAG—erbium-doped yttrium aluminum garnet, NaClO—sodium hypochlorite, NS—normal saline, DW—distilled water, Er,Cr:YSGG—erbium, chromium-doped yttrium, scandium, gallium, and garnet, aPDT—antimicrobial photodynamic therapy, CHX—chlorhexidine, NaOCl—sodium hypochlorite, CFUs—Colony-Forming Units, PIPS—Photon-Induced Photoacoustic Streaming, *P. acnes*—*Propionibacterium acnes*, CLSM—confocal laser scanning microscopy, LIVE/DEAD—LIVE/DEAD viability staining, Nd:YAP—neodymium-doped yttrium aluminum perovskite, ZDB—zone of dead bacteria, TDSC—thermal damage survival curve. * Organisms not stated. SEM used as outcome measure.

### 3.5. Characteristics of Light Sources Used in PDT

Table 6 summarizes the main molecular properties of the Nd:YAG laser in this context. Table 7 presents the key physical properties of the light sources utilized in the studies meeting the inclusion criteria.

**Table 6:** Summary of the properties of Nd:YAG.

Property	Description/Significance
Wavelength	1064 nm (near-infrared).
Chromophore Absorption	Highly absorbed by bacterial pigments (melanin, dark pigments); poor absorption in water and hydroxyapatite.
Mode of Action	Primarily photothermal; energy absorbed by chromophores within bacteria leads to localized heating and bacterial destruction.
Penetration Depth	Effective bactericidal penetration up to 1 mm into dentinal tubules, deeper than many other lasers.
Antibacterial Mechanism	Thermal denaturation of proteins, disruption of bacterial cell membranes, and direct bactericidal effects due to intracellular heating.
Effect on Smear Layer	Causes evaporation, melting, contraction, and recrystallization of smear layer; at higher energies, complete removal and structural changes occur.
Morphological Effects	Melting, glazing, recrystallization, and partial or complete occlusion of dentinal tubules observed.
Thermal Effects	Risk of thermal damage and carbonization at higher energy levels (>3 W); optimal bactericidal effects achieved at controlled parameters (15 Hz, 100 mJ, 1.5 W).
Clinical Usage Recommendation	Used as adjunct to conventional chemical irrigation (NaOCl); does not fully replace chemical disinfection methods.

Source: [38,39,40,41,42,43,44,45,46,47,48].

**Table 7:** Characteristics of Nd:YAG laser used.

**Author and Year**	**Light Source**	**Operating Mode**	**Power Output (mW)**	**Irradiation Time (s)**
Bergmans et al., 2006 [38]	Nd:YAG laser (Smarty A10; DEKA, Firenze, Italy)	15 Hz, short-pulsed mode	1500	4 × 5
Cheng et al., 2012 [39]	Nd:YAG laser (Fontona Lasers, Stegne-7-1210, Ljubljana, Slovenia)	15 Hz, pulsed	1500	4 × 4
Jurić et al., 2016 [40]	Nd:YAG (Fotona, Ljubljana, Slovenia)	15 Hz, pulsed	2000	4 × 5
Kasić et al., 2017 [41]	Nd:YAG (LightWalker, Fotona, Ljubljana, Slovenia)	15 Hz, pulsed	1500	Not specified
Katalinić et al., 2014 [42]	Nd:YAG (Fotona, Ljubljana, Slovenia)	10 Hz, very short-pulsed mode	4000	10
Meire et al., 2012 [43]	Nd:YAG (AT Fidelis, Fotona)	100 Hz, pulsed	15,000	10–120
Moshonov et al., 1995 [44]	Nd:YAG	Not reported	4500	4 × 15
Pirnat et al., 2011 [45]	Nd:YAG (XP-2, Fotona d.d., Slovenia)	Pulsed	Not reported	Sub-second, ms pulses
Schoop et al., 2004 [46]	Nd:YAG (American Dental Technologies, Texas, USA)	10–200 Hz, pulsed	200–5000	0.1
Vatkar et al., 2016 [47]	Nd:YAG (Fotona Fidelis III, Slovenia, Europe)	Continuous mode	1500	5
Wang et al., 2018 [48]	Nd:YAG (Fotona, Ljubljana, Slovenia)	15 Hz, pulsed	1500	15 × 4

## 4. Discussion

### 4.1. Results in the Context of Other Evidence

The neodymium-doped yttrium aluminum garnet (Nd:YAG) laser, operating at a wavelength of 1064 nm, exhibits unique molecular and photothermal properties that make it a valuable tool in root canal disinfection [38,49]. Its high penetration capacity into dentinal tubules allows it to target deeply embedded microbial colonies, especially pigmented bacteria, via rapid thermal effects that denature proteins, disrupt cell membranes, and induce coagulative necrosis [50,51]. This selective absorption by bacterial chromophores enhances its bactericidal potential while sparing surrounding tissue [38,39,40]. Numerous in vitro studies have demonstrated the Nd:YAG laser’s efficacy against key endodontic pathogens. For example, Moritz et al. reported a 99.16% reduction in *Enterococcus faecalis* and *Escherichia coli* populations [52], while Gutknecht et al. achieved a 99.92% reduction in *E. faecalis* using settings of 15 Hz and 100 mJ [53]. Similarly, another study demonstrated up to 99.7% bacterial reduction in root canals, although complete sterilization was rarely attained [38,52,53]. These outcomes highlight the laser’s potential to manage polymicrobial infections, especially when compared to traditional chemical irrigants [47,52,53]. Despite these promising results, limitations remain. The Nd:YAG laser’s bactericidal efficacy is primarily thermal, relying on localized heating to disrupt cell walls and denature intracellular proteins [47,52]. However, its poor absorption in water reduces its effectiveness against non-pigmented bacteria and dense biofilms, often requiring elevated energy densities to achieve meaningful reductions [47,52,54]. Although it can reach bactericidal depths of up to 1000 μm—surpassing the 1100 μm bacterial invasion depth reported in some cases—its effectiveness may be attenuated at these depths, especially in clinical scenarios with persistent biofilms [47,54,55,56,57,58]. Comparative studies suggest that erbium lasers, such as Er:YAG and Er,Cr:YSGG, offer superior bacterial eradication and deeper dentinal tubule penetration than Nd:YAG lasers [39,41,49]. These systems also show greater efficacy in smear layer removal and irrigant activation due to their photoacoustic or photomechanical mechanisms [39,41,59]. In contrast, Nd:YAG relies solely on photothermal interactions, which may be less effective in complex anatomical regions or against mature biofilms. Adjunctive use of Nd:YAG with sodium hypochlorite has shown to improve bacterial clearance and reduce inflammatory markers compared to irrigation alone, offering enhanced clinical outcomes [43]. The laser’s ability to increase push-out bond strength of fiber posts and alter dentin surface properties further underscores its utility [42]. Photothermal effects contribute to smear layer modification and the removal of intracanal medicaments and debris, although these outcomes are often secondary observations and inconsistently quantified [42,44,50]. Sub-millisecond pulsed Nd:YAG modes have emerged as more energy-efficient and bactericidal compared to longer-pulse or continuous-wave settings [46]. Importantly, scanning electron microscopy (SEM) and confocal laser scanning microscopy (CLSM) have confirmed that Nd:YAG can significantly disrupt biofilms, although viable bacteria often persist in deeper canal layers [38,45]. Moreover, temperature rises during laser use have been moderate and not directly correlated with disinfection outcomes, reaffirming the importance of optimizing fluence and pulse parameters rather than relying on heat generation alone [47]. In terms of structural effects, Nd:YAG irradiation induces noticeable dentin alterations such as melting and recrystallization, potentially occluding or exposing dentinal tubules [47,60,61,62,63,64,65,66]. These morphological changes differ substantially from those caused by chemical irrigants like sodium hypochlorite (NaOCl), EDTA, H_2_O_2_, or chlorhexidine (CHX) and may influence the mechanical properties and mineral content of dentin, including the calcium-to-phosphorus ratio—factors relevant to post-treatment restoration [47,50,58]. Clinically, Nd:YAG lasers have shown promising results, especially in cases with significant periapical pathology. Long-term follow-ups have documented favorable outcomes following laser-enhanced disinfection, particularly when optimal settings (e.g., 15 Hz, 100 mJ, 1.5 W) and careful fiber optic tip movement are employed [47,53,56,59,67,68]. Nevertheless, studies agree that the laser should serve as a complementary tool rather than a standalone solution, as it has not demonstrated superiority over conventional irrigants such as NaOCl [29,38,60]. This conclusion is further reinforced by recent reviews. Dawasaz et al. (2022) found that diode lasers could significantly reduce microbial counts in vivo, although protocol heterogeneity and limited long-term data impeded strong clinical recommendations [66]. Fransson et al. (2013) reported improved microbial reductions with laser-assisted chemomechanical preparation but could not confirm consistent benefits in periapical healing due to methodological variability [59]. Similarly, Hazrati et al. (2024) suggested that laser-assisted therapy may enhance periapical healing, yet high-quality randomized trials remain scarce [67]. Despite its strengths, the Nd:YAG laser’s reduced efficacy against non-pigmented bacteria and mature biofilms, along with its inability to achieve complete sterilization, remain critical limitations [52,55,62,63]. Therefore, future research should focus on refining laser parameters, exploring hybrid modalities such as photodynamic therapy (PDT) and laser-activated irrigation (LAI), and advancing the understanding of laser–biofilm interactions at the molecular level [51,52]. These innovations may further enhance the clinical efficacy and integration of Nd:YAG lasers in endodontic disinfection protocols [51,52,68,69,70,71].

### 4.2. Limitations of the Evidence

The current body of evidence on the efficacy of Nd:YAG lasers in root canal disinfection is constrained by several key limitations. Most notably, the overwhelming reliance on in vitro studies limits the generalizability of findings to clinical practice, as these controlled laboratory settings do not accurately replicate the anatomical, biological, and microbial complexities of the human oral environment. Additionally, there is considerable variability in experimental protocols, including differences in the laser settings, exposure times, fiber types, and microbial strains used. This heterogeneity hampers direct comparison between studies and undermines the ability to draw consistent conclusions. Moreover, many studies lack standardized outcome measures, and few assess long-term antimicrobial effects or potential microbial recolonization, which are critical for evaluating clinical relevance. The limited number of high-quality in vivo investigations and randomized controlled trials further weakens the strength of the evidence. These gaps highlight the urgent need for more rigorous, standardized, and clinically oriented research to fully elucidate the therapeutic value of Nd:YAG lasers in endodontic disinfection.

### 4.3. Limitations of the Review Process

Despite providing a comprehensive synthesis of the existing literature, this systematic review has several noteworthy limitations. Firstly, all included studies were either in vitro or ex vivo, limiting the direct applicability of findings to clinical practice due to the absence of complex biological interactions present in in vivo settings. Secondly, significant heterogeneity in study protocols—such as variations in Nd:YAG laser parameters, microbial species tested, sample preparation, and disinfection outcome measures—makes it difficult to compare results across studies or conduct meaningful meta-analyses. Thirdly, while the included studies were assessed as having low risk of bias, their overall methodological diversity and lack of long-term follow-up reduce confidence in their durability and reproducibility. Additionally, the exclusion of non-English publications and gray literature introduces potential language and publication bias, possibly omitting relevant findings. Finally, the limited number of clinical trials in the evidence base highlights the need for more robust, standardized, and clinically focused research to better define the efficacy and practical integration of Nd:YAG laser therapy in endodontic disinfection.

A quantitative meta-analysis was not performed in this review due to substantial heterogeneity among the included studies. The sources of heterogeneity included the following: (1) variations in experimental design; (2) diverse microbial targets, with some studies evaluating mono-species infections and others assessing mixed-species or polymicrobial biofilms; (3) inconsistent Nd:YAG laser parameters such as wavelength calibration, power output, irradiation duration, and pulse frequency; (4) differing control groups and disinfection comparators across studies; and (5) lack of uniform outcome measures for microbial viability or reduction (e.g., CFU counts, SEM analysis, CLSM, ZDB). Due to these methodological discrepancies, data pooling would not yield valid or interpretable summary estimates, and meta-analytic synthesis was deemed inappropriate. Instead, a qualitative synthesis was conducted to preserve the integrity of the individual study contexts and findings.

### 4.4. Implications for Practice, Policy, and Future Research

The findings from this systematic review on the efficacy of Nd:YAG lasers in root canal disinfection have significant implications for clinical practice, policy, and future research. Clinically, the Nd:YAG laser shows considerable promise as an adjunctive treatment for root canal disinfection, especially in cases involving persistent biofilms and challenging anatomical structures like lateral canals and dentinal tubules. Dental practitioners are encouraged to integrate Nd:YAG laser treatment alongside traditional chemical irrigants, such as sodium hypochlorite and chlorhexidine, to improve disinfection outcomes and reduce treatment failure rates associated with resilient pathogens, particularly Enterococcus faecalis. From a policy perspective, the current absence of standardized clinical protocols highlights the urgent necessity for developing consensus guidelines that detail precise laser operation parameters, such as optimal pulse durations, power settings, and irradiation techniques. This standardization is essential to ensure consistent, safe, and effective clinical implementation. Regarding future research, it is imperative to conduct robust, randomized controlled clinical trials to validate the laboratory findings, investigate the long-term sustainability of antimicrobial effects, assess potential complications or adverse events, and compare the efficacy of Nd:YAG lasers with alternative laser systems and conventional endodontic treatments. Additionally, further investigation into the molecular mechanisms underlying the laser’s bactericidal effects could provide valuable insights, contributing to more effective and targeted therapeutic strategies.

While the Nd:YAG laser demonstrates significant antimicrobial effects in vitro, a major limitation is the absence of clinical evidence confirming its impact on long-term endodontic treatment outcomes. Specifically, no high-quality studies have shown that laser-assisted disinfection improves healing rates, reduces treatment failure, or enhances patient-reported outcomes compared to conventional protocols. The current evidence base is predominantly composed of laboratory-based research with limited translational value. As such, the therapeutic benefit of Nd:YAG lasers in routine endodontic practice remains hypothetical until validated by rigorously designed clinical trials.

## 5. Conclusions

The Nd:YAG laser shows promising antimicrobial efficacy in root canal disinfection, particularly due to its deep penetration and photothermal effects. While in vitro studies report significant bacterial load reductions, complete biofilm eradication remains inconsistent, and Er:YAG or Er,Cr:YSGG lasers often demonstrate superior outcomes. However, the current evidence is limited by methodological heterogeneity, lack of standardized protocols, and absence of clinical trials confirming improved treatment outcomes. As such, the Nd:YAG laser should be considered an adjunct to, not a replacement for, conventional irrigation. Future research should focus on well-designed clinical trials, standardized laser parameters, and molecular studies to clarify the laser’s clinical utility and optimize its application in endodontic practice.

## Figures and Tables

**Table 1:** Search syntax used in this study.

Source	Search Term	Number of Results
PubMed	(“Nd:YAG” [Title/Abstract] OR “neodymium:YAG” [Title/Abstract] OR “Nd:YAG laser” [Title/Abstract]) AND (“root canal disinfection” [Title/Abstract] OR “endodontic disinfection” [Title/Abstract] OR “root canal therapy” [Title/Abstract]) AND (disinfection [Title/Abstract] OR sterilization [Title/Abstract] OR “microbial reduction” [Title/Abstract])	14
Embase	(‘nd:yag’:ab,ti OR ‘neodymium:yag’:ab,ti OR ‘nd:yag laser’:ab,ti) AND (‘root canal disinfection’:ab,ti OR ‘endodontic disinfection’:ab,ti OR ‘root canal therapy’:ab,ti) AND (disinfection:ab,ti OR sterilization:ab,ti OR ‘microbial reduction’:ab,ti)	17
Scopus	(TITLE-ABS(“Nd:YAG”) OR TITLE-ABS(“neodymium:YAG”) OR TITLE-ABS(“Nd:YAG laser”)) AND (TITLE-ABS(“root canal disinfection”) OR TITLE-ABS(“endodontic disinfection”) OR TITLE-ABS(“root canal therapy”)) AND (TITLE-ABS(disinfection) OR TITLE-ABS(sterilization) OR TITLE-ABS(“microbial reduction”))	17
Cochrane	(“Er:YAG laser”:ti,ab OR “erbium:YAG laser”:ti,ab) AND (disinfection:ti,ab OR antibacterial:ti,ab OR bactericidal:ti,ab) AND (efficacy:ti,ab OR effectiveness:ti,ab) AND (bacteria:ti,ab OR microbial:ti,ab OR microbiological:ti,ab)	0

**Table 2:** Selection criteria used in this study.

Inclusion Criteria	Exclusion Criteria
In vitro studies investigating the antimicrobial or bactericidal effects of the Nd:YAG laser. Research evaluating microbial susceptibility to Nd:YAG laser treatment across various bacterial or fungal species. Studies in which the Nd:YAG laser serves as the primary intervention within the disinfection protocol. Investigations assessing potential synergistic effects of combining Nd:YAG laser therapy with conventional antimicrobial agents. Studies employing controlled experimental designs, including comparisons with untreated samples, placebo groups, or other antimicrobial technologies. Studies comparing the efficacy of Nd:YAG laser disinfection with standard antimicrobial methods in terms of microbial load reduction or eradication.	Non-peer-reviewed literature, including conference abstracts, case reports, editorials, opinion pieces, book chapters, and unpublished theses. Studies not meeting minimum standards of scientific rigor or lacking detailed methodological reporting. Articles published in languages other than English. Duplicate publications or multiple articles derived from the same dataset without additional or novel findings. Research focused on non-microbial applications of the Nd:YAG laser. Studies lacking a control or comparison group to contextualize antimicrobial outcomes. Studies examining other laser types. Pathogens not relevant to endodontic infections. In vitro studies using highly artificial models with limited clinical translatability or real-world relevance.

**Table 3:** The results of the quality assessment and risk of bias across the studies.

Study	1	2	3	4	5	6	7	8	9	10	Total	Risk
Bergmans et al., 2006 [38]	1	1	1	1	1	1	1	1	1	0	9	Low
Cheng et al., 2012 [39]	1	1	1	1	1	1	1	1	1	1	10	Low
Jurić et al., 2016 [40]	1	1	1	1	1	1	1	1	1	1	10	Low
Kasić et al., 2017 [41]	1	1	1	1	1	1	1	1	1	0	9	Low
Katalinić et al., 2014 [42]	1	1	1	1	1	1	1	1	1	1	10	Low
Meire et al., 2012 [43]	1	1	1	1	1	1	1	1	1	1	10	Low
Moshonov et al., 1995 [44]	1	1	1	1	1	1	1	1	0	1	9	Low
Pirnat et al., 2011 [45]	1	1	1	1	1	1	1	1	1	1	10	Low
Schoop et al., 2004 [46]	1	1	1	1	1	1	1	1	0	1	8	Low
Vatkar et al., 2016 [47]	1	1	1	1	0	1	1	1	1	1	8	Low
Wang et al., 2018 [48]	1	1	1	1	0	1	1	1	0	1	7	Low

## Data Availability

No new data were created or analyzed in this study.
